# The effect of sex, season and gametogenic cycle on gonad yield, biochemical composition and quality traits of *Paracentrotus lividus* along the North Atlantic coast of Portugal

**DOI:** 10.1038/s41598-019-39912-w

**Published:** 2019-02-28

**Authors:** Filipa Rocha, Luís F. Baião, Sara Moutinho, Bruno Reis, Ana Oliveira, Francisco Arenas, Margarida R. G. Maia, António J. M. Fonseca, Manuela Pintado, Luisa M. P. Valente

**Affiliations:** 1 CIIMAR/CIMAR, Interdisciplinary Centre of Marine and Environmental Research, University of Porto, Terminal de Cruzeiros do Porto de Leixões, Av. General Nórton de Matos, S/N, 4450-208 Matosinhos, Portugal; 2 ICBAS, Abel Salazar Biomedical Sciences Institute, University of Porto, Rua Jorge Viterbo Ferreira 228, 4050-313 Porto, Portugal; 3 CBQF, Faculty of Biotechnology, Portuguese Catholic University, Rua Dr. António Bernardino de Almeida, 4200-072 Porto, Portugal; 4 REQUIMTE, LAQV, ICBAS, Abel Salazar Biomedical Sciences Institute, University of Porto, Rua de Jorge Viterbo Ferreira 228, 4050-313 Porto, Portugal

## Abstract

Sea urchin population harvest in the North Atlantic coast of Portugal was characterized in terms of gonad yield, nutritional composition and important market-related traits, over one reproductive cycle (March 2016 to March 2017). Most of the quality attributes showed a seasonal variation strongly dependent on sea urchin sex. Maximum gonad yield (18%) was observed in March 2017. A single spawning event occurred between May and July. Gonads are rich sources of protein (12–18% WW) with low fat content (≤6% WW), that increase during the gametogenic stages of recovery and growing (November-December). Polyunsaturated fatty acids were the dominant class in both sexes (4.2–14.7 mg.g^−1^ WW), being preferentially accumulated in females. Total gonads carotenoid varied seasonally, with the highest level being observed in males during spawning season. Echinenone was the main pigment present in gonads, showing highest concentrations in males during spawning and gonad recovering. During the growing and early maturation period gonads were more reddish, yellowish and brighter, as well as more firm, irrespectively of the sex. Based on all seasonal changes affecting gonad yield and quality, the period between November and February seems the most suitable to harvest high quality gonads in the Atlantic coast of Portugal.

## Introduction

Sea urchin gonads (roe) are a prized and gourmet seafood delicacy, due to its unique flavour^1^. The global volume of sea urchin captures in 2016 reached 28575 tonnes, whilst farming has further contributed with 10057 tonnes^2^. Japan is an important driver of sea urchin market and represents 75% of total imports (11116 tonnes) of major sea urchin product forms (live, fresh or chilled, frozen and dried, salted, or brined) estimated in 14739 tonnes in 2016^2^. Urchin roe can reach a high commercial value as demand often exceeds the market supply. Recently, excessive exploitation and destructive harvesting methods have caused the depletion of wild stocks^3^.

The sea urchin *Paracentrotus lividus* has being intensively harvested in most of its geographical range, throughout the Mediterranean Sea and the North eastern Atlantic, from Scotland (northern) to Canary Islands (southern)^4^. In Europe, the natural stocks found in France, Italy and Spain are considered overexploited, even so intensive harvesting is still occurring: in the North regions of Spain landings reached 520 tonnes in 2014^5^. In Portugal, *P*. *lividus* is the dominant echinoid species in the rocky shores^6^ and landings are mainly concentrated in the North Atlantic coast (Viana do Castelo) where harvests reached 28 tonnes in 2015^7^. Commercial harvesting has increased over the last decade, which might reflect the demand to supply markets of nearby regions, such as North Spain^8^.

The main market for sea urchin gonads is Japan where the price for fresh gonads is considerable higher than for frozen or dried/salted; fresh roe can reach 40–100€/kg^9^. A premium gonad is characterized by its size combined with specific sensory traits sought by the consumers (taste, colour, texture and freshness). Desirable gonads present a bright yellowish-orange colour^1,10^, *umami* taste^11^, firm texture and high freshness^12^. However, gonads size and sensory traits largely depend on several factors, namely: (i) sea urchin species, (ii) gonads maturation stage (reproductive cycle), (iii) environmental cues (water temperature, photoperiod) and (iv) animal nutritional status (food availability and nutritional value).

*P*. *lividus* reproduction follows a predictable seasonal pattern characterized by six gametogenic stages^13^. The annual reproductive cycle of this species can present one or two spawning seasons, depending on the habitat, latitudinal location and environmental conditions to which populations are subjected to^4^. Some studies have investigated the role of exogenous factors, such as temperature, photoperiod and primary production in sea urchin gametogenesis and spawning. However, it has been suggested that the environmental cues that trigger spawning may differ from those that stimulate gonadal growth and development^14^. Byrne^13^ observed that temperatures lower than 13 °C may inhibit spawning of *P*. *lividus* populations in Ireland. Shpigel, *et al*.^15^ reported that short days and increasing temperatures (18–22 °C) enhanced gonad development and gametogenesis, while Spirlet, *et al*.^16^, Spirlet, *et al*.^17^ suggested that the spawning event may be triggered by shorter day-length. Latitudinal patterns can also be found in echinoids reproductive cycles, the gonad index of *P*. *lividus* showed a clear geographical trend with the Atlantic populations reaching higher GSI than the Mediterranean populations^18^.

During the reproductive cycle, gonads from both females and males increase in size (yield) over two distinct periods: before gametogenesis, when nutrient reserves are stored in the nutritive phagocytes, and during gametogenesis, when increased number and size of gametes is observed^14^. Gonads are preferentially consumed before the beginning of gametogenesis, when their nutritive phagocytes are in maximum volume and rich in nutrients, attributes that contribute to a sweeter flavour and firmer texture. Once gametogenesis begins, the taste and texture of gonads deteriorates, mainly due to the utilization of yolk protein into synthesis of new compounds^19^.

Gonad colour is a pivotal factor determining its quality and marketability. The desirable yellow-orange colour is determined by carotenoids deposited in the gonads, mainly the accumulation of echinenone that is its dominant pigment^10^. Gonad echinenone content is limited by the availability, uptake and bioconversion of β-carotene from dietary sources, in particular from algae^20,21^. The consumption of different algae with different carotenoid profiles influences the concentration of pigments present in sea urchins^22^. Also, diet type (natural or formulated) can drastically influence not only the biochemical composition of gonads but also their organoleptic properties, such as flavour and texture^11,23^.

To date, apart from a few studies focusing environmental^24^, ecological^6,25^ and conservational^8^ issues of Portuguese populations of *P*. *lividus*, little is known about important market-related traits of this high valued species and its potential to commercialization. This study aims to characterize the seasonal variations of *P*. *lividus* gonads in terms of maximum yield, nutritional value, colour and texture over one reproductive cycle. The possible relationship between sex, season and gametogenic cycle and selected market-related traits was discussed, for the first time in a wild population, and the best period to harvest high quality gonads in the North Atlantic coast of Portugal was determined.

## Results

### Environmental condition

**Figure 1:**
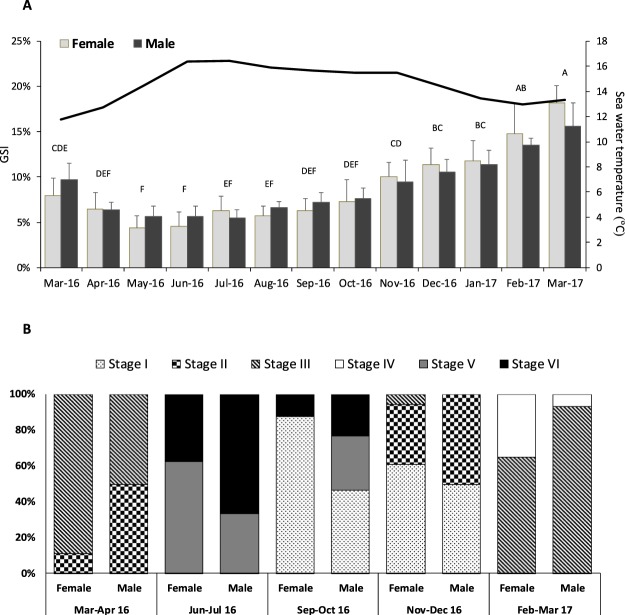
Annual reproductive cycle of females and males *P*. *lividus* in Praia Norte (Portugal); (**A**) variations in the gonad somatic index (GSI) over a 13-month period; (**B**) Gametogenic stages of ovary and testis through histological examination. Values are mean ± standard deviation; per month the 18 individuals were pooled by sex: N = 3 pools of female gonads and N = 3 pools of male gonads were established. Superscripts in capital letters (^ABC^) indicate significant differences between months (P < 0.05).

Detailed data on sea water temperature was obtained through daily records of an oceanic buoy located close to our study area. The annual variation of water temperature was minor and ranged between 11.8–16.4 °C (Fig. 1A). Temperature steadily rose from the lowest value recorded in March 2016 (11.8 °C) to its maximum (16.4 °C) observed during June-July 2016. Then, it slowly decreased until reaching 13 °C in February 2017. Records of day length indicated a seasonal variation, with a gradual increase of light hours from winter to summer season, followed by a decrease afterwards. Similar day lengths were registered in march-16 (12 h:12 m) and march-17 (11 h:47 m)^26^

### Gonad index and gametogenic stages

Total body weight (105 ± 17.1 g) and test diameter (6.4 ± 0.4 cm) of all sampled animals were not significantly different over time (*P* ≥ 0.05). This evidences a size-class consistency in the evaluated sea urchin groups, allowing a direct comparison of gonadal quality traits over the 13-month period. Gonad weight decreased to almost half of its value from March-16 to June (10.3 ± 2.7 g to 5.5 ± 2.3 g, respectively), then slowly increased until March-17 (15 ± 3.3 g), consequently affecting GSI values over the year. The annual reproductive cycle of *P*. *lividus* population from Praia Norte is presented in Fig. 1A. The GSI varied significantly over the 13-month period (*P* < 0.05), but was similar between sexes (*P* ≥ 0.05, Supplementary Table S1). GSI decreased from March 2016 (9%) to a minimum level of 5% in May-June; then values steadily increase until March of the following year, when they peaked (18% for females and 15.6% for males). Changes on the GSI reflected gonads maturation stage (Fig. 1B). During March-April 2016, females and 50% of males presented gonads at stage III (pre-mature), indicating the beginning of gametogenesis. From June to July 2016 spawning occurred; most of the sea urchins presented empty gonads at the partly spawned (V) or spent (VI) stages. The recovery (I) and growing (II) stages of gonads were more noticeable from November to December 2016, in both sexes. During this period, it was possible to identify a large amount of nutritive phagocytes as well as the presence of primary gametes in the gonads. The onset of gonads growth (November) was closely followed by an increase of gonad size and volume (GSI), that peaked in March 2017, when maturation was undergoing and mature gametes were accumulated in gonads (stage III and IV).

### Biochemical composition

**Figure 2:**
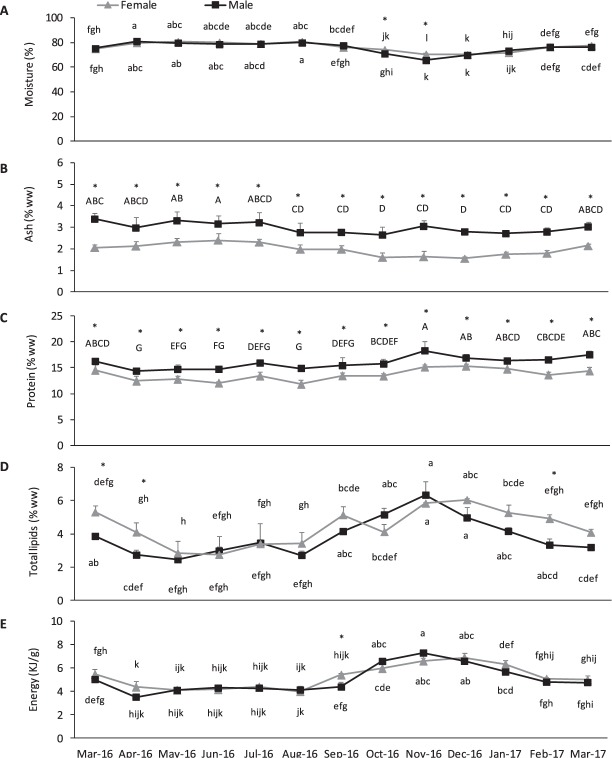
Changes in (**A**) moisture (%), (**B**) ash, (**C**) protein, (**D**) total lipids (% WW) and (**E**) energy (KJ/g WW) contents in gonads (ovary and testis) of *P*. *lividus* collected at Praia Norte (Portugal) over a 13-month period. Values are mean ± standard deviation; per month the 18 individuals were pooled by sex: N = 3 pools of female gonads and N = 3 pools of male gonads were established. Superscripts in capital letters (^ABC^) indicate differences between months, asterisk (*) indicate differences between sexes and superscripts in lowercase letters indicate significant differences between sex X month (^abc^) (P < 0.05).

The nutritional value of sea urchin gonads is presented in Fig. 2. All analysed parameters were significantly affected by sex and time (*P* ≥ 0.05, Supplementary Table S1). Protein was the major component of gonads for both male and female *P*. *lividus* (Fig. 2C). Males had significantly higher protein content (14–18% WW) than females (12–15% WW). Both sexes showed highest protein content in November, during gonad growing (stage II) (*P* < 0.05). Lipid content of gonads (Fig. 2D) showed a significant interaction between sex and time (*P* < 0.05, Supplementary Table S1). Likewise proteins, the highest lipid content (6%) was observed in November, during gonad growing (stage II). Minimum levels (2–3% WW) were achieved at the beginning of the spawning season, in May-June, when gonads were mostly spent (stage VI). Energy content in gonads (Fig. 2E) followed a similar pattern of total lipids, with both main factors interacting significantly (*P* ≥ 0.05). Again, gonad energy was at minimum levels during spawning (3–4 kJ/g WW), and achieved maximum content (7 kJ/g WW) in November, during growing. Both sexes showed similar moisture content, ranging from 66 to 81% over the year (Fig. 2A) however females had significantly higher moisture during October and November than males. Moisture was highly correlated with the lipid content (r = 0.97, Table 1). Ash content was higher in male (2.7–3.4% WW) than in female (1.6–2.4% WW) gonads (*P* < 0.05) and peaked in June during spawning (Fig. 2B).

**Table 1:** Values of Pearson’s correlation coefficients between yield, biochemical composition and colour determination (carotenoids and CIE parameters) of *P*.

			1	2	3	4	5	6	7	8	9	10	11	12
Yield (%) and Biochemical composition (%WW)	(1)	GSI												
	(2)	Moisture												
	(3)	Protein		−0.69**										
	(4)	Lipids		−0.97**										
Fatty acids (mg.g-1 DM)	(5)	ARA								−0.58**				
	(6)	EPA				0.67**				−0.48*	0.61*			
	(7)	DHA				0.45*								
Colour parameter (CIE)	(8)	L*	0.63**		0.41*									
	(9)	a*				0.75**								
	(10)	b*	0.66**	−0.54**		0.72**					0.77**			
Carotenoid analysis (µg.g-1 WW)	(11)	Total carotenoid	−0.45*							−0.52*		−0.39*		
	(12)	β-carotene					0.40*	0.40**		−0.36*			0.71**	
	(13)	Echinenone			0.52**								0.77**	0.72**

*lividus* gonads determined for a 5-month period. N = 30, for the five months analysed: April, June, September and November 2016 and February 2017. **P < 0.01 (two-tailed) and *P < 0.05 (two-tailed); bold values indicate negative correlations.

The fatty acid (FA) composition of female and male gonads of *P*. *lividus* is shown in Table 2. In both sexes, the polyunsaturated fatty acids (PUFA) were the dominant FA class (4.2–14.7 mg.g^−1^ WW), followed by saturated fatty acids (SFA) (2.3–11.0 mg.g^−1^ WW) and monounsaturated fatty acids (MUFA) (2.2–7.7 mg.g^−1^ WW). The most represented PUFA was 20:5n-3 (EPA), varying between 1.5–4.8 mg.g^−1^ WW followed by 20:4n-6 (ARA; 1.2–3.7 mg.g^−1^ WW), 18:4n-3 (0.1–2.5 mg.g^−1^ WW) and 18:3n-3 (ALA, 0.08–0.85 mg.g^−1^ WW). Reduced amounts of 22:6n-3 (DHA; 0.02–0.11 mg.g^−1^ WW) were found in gonads of both sexes. Females had significantly higher levels of PUFA than males, particularly from the n-3 family (*P* < 0.05), however both DHA and ARA levels were similar between sexes (*P* ≥ 0.05). As a reference for the consumption of seafood as part of a healthy diet, the levels of EPA + DHA in fresh gonads varied between 1.5–4.9 mg/g, being highest in November for both sexes. Most PUFA showed a seasonal variation in their content, being generally higher in November during gonad growth (stage II). Among SFA, the main compounds were 16:0 (1.3–6.2 mg.g^−1^ WW), 14:0 (0.5–3.3 mg.g^−1^ WW) and 18:0 (0.3–0.7 mg.g^−1^ WW). Total SFA were significantly higher in females than males, and in November compared to the other months (*P* < 0.05). MUFA were mainly represented by 20:1n-9 (0.6–1.8 mg.g^−1^ WW), 20:1n-11 (0.5–1.5 mg.g^−1^ WW) and 18:1n-7 (0.3–1.2 mg.g^−1^ WW), which were in higher amount in female gonads and preferentially concentrated in November (growing stage of gonads) (*P* < 0.05). A positive correlation was found between total lipids and EPA and DHA levels (r = 0.67 and 0.45, respectively). EPA and ARA were negatively correlated to L* (r = −0.58 and −0.48) and positively correlated to β-carotene (r = 0.40, Table 1).

**Table 2:** Gonad fatty acid composition (mg.g^−1^ WW) of *P*.

Sex	Female					Male					Two-way ANOVA *P*-value		
Month	Apr-16	Jun-16	Sep-16	Nov-16	Feb-17	Apr-16	Jun-16	Sep-16	Nov-16	Feb-17	Sex	Month	X
14:0	2.4 ± 0.6^B^*	3.0 ± 0.5^B^*	2.7 ± 0.4^B^*	3.3 ± 0.3^A^*	2.2 ± 1.1^B^*	0.5 ± 0.1	1.3 ± 0.4	1.0 ± 0.3	2.8 ± 0.7	0.6 ± 0.1	<0.01	<0.01	0.2
16:0	3.5 ± 1.0^B^*	4.9 ± 0.5^B^*	4.9 ± 1.0^B^*	6.2 ± 0.3 ^A^*	4.0 ± 2.0^B^*	1.2 ± 0.2	2.8 ± 0.9	2.3 ± 0.4	5.4 ± 1.4	1.5 ± 0.3	<0.01	<0.01	0.6
18:0	0.4 ± 0.1^C^	0.5 ± 0.04^BC^	0.8 ± 0.3^AB^	0.8 ± 0.1^A^	0.5 ± 0.2^C^	0.3 ± 0.05	0.5 ± 0.1	0.6 ± 0.1	0.8 ± 0.1	0.3 ± 0.1	0.2	<0.01	0.6
20:0	0.2 ± 0.01 ^C^*	0.2 ± 0.01^BC^*	0.3 ± 0.1^AB^*	0.3 ± 0.01 ^A^*	0.2 ± 0.1^BC^*	0.1 ± 0.01	0.1 ± 0.03	0.2 ± 0.01	0.3 ± 0.1	0.1 ± 0.01	<0.01	<0.01	0.4
Σ SFA	6.8 ± 1.8^B^*	8.9 ± 1.1^B^*	9.1 ± 1.8^AB^*	11.0 ± 0.4 ^A^*	7.2 ± 3.7^B^*	2.3 ± 0.5	5.0 ± 1.6	4.3 ± 0.8	9.7 ± 2.3	2.6 ± 0.6	<0.01	<0.01	0.4
16:1n-7	0.6 ± 0.2^B^*	1.0 ± 0.2 ^AB^*	0.8 ± 0.1 ^AB^*	1.1 ± 0.2^A^*	0.8 ± 0.4^B^*	0.1 ± 0.02	0.4 ± 0.2	0.3 ± 0.1	0.9 ± 0.3	0.2 ± 0.1	<0.01	<0.01	0.5
18:1n-9	0.4 ± 0.1^B^*	0.6 ± 0.2^AB^*	0.4 ± 0.1^B^*	0.6 ± 0.1 ^A^*	0.4 ± 0.2^B^*	0.1 ± 0.05	0.3 ± 0.1	0.2 ± 0.04	0.6 ± 0.1	0.2 ± 0.1	<0.01	<0.01	0.3
18:1n-7	0.7 ± 0.2^B^*	0.9 ± 0.1^B^*	1 ± 0.2^AB^*	1.2 ± 0.1 ^A^*	0.7 ± 0.3^B^*	0.3 ± 0.03	0.6 ± 0.2	0.5 ± 0.1	1.1 ± 0.3	0.3 ± 0.04	<0.01	<0.01	0.3
20:1n-11	1.1 ± 0.2^BC^*	1.5 ± 0.1 ^A^*	1.3 ± 0.2^AB^*	1.5 ± 0.1^A^*	0.9 ± 0.4 ^C^*	0.6 ± 0.1	1.1 ± 0.3	0.8 ± 0.1	1.4 ± 0.3	0.5 ± 0.1	<0.01	<0.01	0.3
20:1n-9	1.2 ± 0.3^BC^*	1.7 ± 0.3^AB^*	1.3 ± 0.3^BC^*	1.7 ± 0.2^A^*	1.2 ± 0.6^C^*	0.7 ± 0.05	1.4 ± 0.4	0.9 ± 0.1	1.8 ± 0.6	0.6 ± 0.1	<0.01	<0.01	0.6
22:1n-9	0.7 ± 0.2^B^*	0.8 ± 0.1 ^AB^*	0.7 ± 0.2^B^*	0.8 ± 0.1^A^*	0.5 ± 0.3^B^*	0.2 ± 0.03	0.6 ± 0.1	0.4 ± 0.1	0.9 ± 0.2	0.2 ± 0.03	<0.01	<0.01	0.1
Σ MUFA	5.3 ± 1.3^B^*	7.1 ± 0.9^AB^*	6.2 ± 1.2^B^*	7.7 ± 0.5^A*^	5.1 ± 2.5^B^*	2.2 ± 0.2	4.8 ± 1.4	3.7 ± 0.7	7.5 ± 1.9	2.2 ± 0.3	<0.01	<0.01	0.4
18:2n-6 (LA)	0.2 ± 0.1^B^*	0.3 ± 0.1^B^*	0.3 ± 0.1^B^*	0.4 ± 0.01 ^A^*	0.2 ± 0.1^B^*	0.1 ± 0.01	0.1 ± 0.1	0.1 ± 0.02	0.4 ± 0.1	0.1 ± 0.03	<0.01	<0.01	0.2
18:3n-6	0.05 ± 0.01^B^*	0.1 ± 0.01^B^*	0.1 ± 0.01^B^*	0.1 ± 0.01 ^A^*	0.1 ± 0.03^B^*	0.01 ± 0.01	0.04 ± 0.02	0.03 ± ± 0.01	0.1 ± 0.02	0.02 ± 0.01	<0.01	<0.01	0.3
18:3n-3 (ALA)	0.3 ± 0.1^B^*	0.5 ± 0.1^B^*	0.8 ± 0.2 ^A^*	0.8 ± 0.01 ^A^*	0.4 ± 0.3^B^*	0.1 ± 0.02	0.2 ± 0.1	0.2 ± 0.01	0.7 ± 0.2	0.2 ± 0.04	<0.01	<0.01	0.2
18:4n-3	0.6 ± 0.1^B^*	1.1 ± 0.1^B^*	2.5 ± 0.6 ^A^*	2.5 ± 0.2^A^*	1.1 ± 0.8^B^*	0.1 ± 0.04	0.4 ± 0.2	0.7 ± 0.3	2.0 ± 0.5	0.5 ± 0.1	<0.01	<0.01	0.1
20:2n-6	0.2 ± 0.1^BC^	0.3 ± 0.1^AB^	0.3 ± 0.1^BC^	0.4 ± 0.01^A^	0.2 ± 0.1^C^	0.3 ± 0.03	0.4 ± 0.1	0.4 ± 0.04	0.6 ± 0.1	0.2 ± 0.01	0.05	<0.01	0.3
20:3n-6	0.1 ± 0.02^B^	0.1 ± 0.02^AB^	0.1 ± 0.01^B^	0.1 ± 0.01^A^	0.1 ± 0.04^B^	0.1 ± 0.01	0.1 ± 0.03	0.1 ± 0.01	0.2 ± 0.04	0.1 ± 0.01	0.2	<0.01	0.6
20:4n-6 (ARA)	1.8 ± 0.4^C^	2.2 ± 0.2^BC^	3.0 ± 0.6^AB^	3.2 ± 0.3^A^	1.9 ± 1.2^C^	1.5 ± 0.2	2.4 ± 0.6	2.2 ± 0.3	3.7 ± 0.6	1.2 ± 0.1	0.3	<0.01	0.4
20:3n-3	0.3 ± 0.1^C^	0.5 ± 0.1^BC^	0.8 ± 0.2^AB^	0.9 ± 0.1^A^	0.4 ± 0.2^C^	0.2 ± 0.05	0.5 ± 0.1	0.6 ± 0.1	1 ± 0.2	0.3 ± 0.04	0.3	<0.01	0.6
20:4n-3	0.2 ± 0.03^B^*	0.3 ± 0.01^B^*	0.7 ± 0.2^A^*	0.8 ± 0.1^A^*	0.3 ± 0.2^B^*	0.1 ± 0.02	0.3 ± 0.1	0.4 ± 0.1	0.7 ± 0.1	0.2 ± 0.05	0.02	<0.01	0.5
20:5n-3 (EPA)	2.2 ± 0.6^C^*	2.6 ± 0.2^BC^*	4.4 ± 0.8^AB^*	4.8 ± 0.4^A^*	2.9 ± 1.7^C^*	1.5 ± 0.2	2.3 ± 0.6	2.6 ± 0.4	4.8 ± 0.5	1.7 ± 0.2	0.01	<0.01	0.4
22:4n-6	0.1 ± 0.01^bc^	0.1 ± 0.01^bc^	0.1 ± 0.01^bc^	0.1 ± 0.01^bc^	0.04 ± 0.02^c^	0.05 ± 0.01^bc^	0.1 ± 0.02^ab^	0.1 ± 0.01^ab^	0.1 ± 0.02^a^	0.04 ± 0.01^c^	<0.01	<0.01	0.04
22:5n-3	0.1 ± 0.02^c^	0.1 ± 0.01^bc^	0.1 ± 0.02^bc^	0.2 ± 0.01^bc^	0.1 ± 0.05^c^	0.1 ± 0.03^bc^	0.2 ± 0.04^ab^	0.2 ± 0.03^abc^	0.3 ± 0.04^a^	0.1 ± 0.01^c^	<0.01	<0.01	0.04
22:6n-3 (DHA)	0.05 ± 0.01^B^	0.05 ± 0.01^B^	0.05 ± 0.01^B^	0.1 ± 0.03^A^	0.04 ± 0.01^B^	0.03 ± 0.01	0.1 ± 0.02	0.05 ± 0.01	0.1 ± 0.04	0.02 ± 0.01	0.9	<0.01	0.3
Σ PUFA	6.3 ± 1.5^C^*	8.4 ± 0.9^BC^*	13.3 ± 2.9^AB^*	14.5 ± 1.2 ^A*^	7.9 ± 4.9^C^*	4.2 ± 0.7	7.2 ± 2.1	7.8 ± 1.2	14.7 ± 2.4	4.6 ± 0.6	0.02	<0.01	0.4
EPA + DHA	2.3 ± 0.6^C^*	2.7 ± 0.2^BC^*	4.5 ± 0.8^AB*^	4.9 ± 0.4^A^*	2.9 ± 1.7^C^*	1.5 ± 0.2	2.4 ± 0.6	2.6 ± 0.4	4.9 ± 0.6	1.8 ± 0.2	0.01	<0.01	0.4
Σ n-6	2.4 ± 0.6^B^	3.1 ± 0.5^B^	3.7 ± 0.8^AB^	4.3 ± 0.3^A^	2.6 ± 1.5^B^	1.9 ± 0.3	3.2 ± 0.9	2.9 ± 0.3	5.0 ± 0.9	1.6 ± 0.2	0.3	<0.01	0.4
Σ n-3	3.8 ± 0.9^B^*	5.2 ± 0.5^B^*	9.5 ± 2.1^A^*	10.1 ± 0.9^A^*	5.2 ± 3.2^B^*	2.2 ± 0.4	4.0 ± 1.1	4.9 ± 0.9	9.6 ± 1.6	3.0 ± 0.5	<0.01	<0.01	0.30
n-6/n-3	0.6 ± 0.01^b^	0.6 ± 0.03^b^	0.4 ± 0.01^c^	0.4 ± 0.01^bc^	0.5 ± 0.03^bc^	0.8 ± 0.1^a^	0.8 ± 0.01^a^	0.6 ± 0.04^b^	0.5 ± 0.05^bc^	0.5 ± 0.03^bc^	<0.01	<0.01	<0.01

*lividus* females and males collected at Praia Norte (Portugal) at five distinct periods. Values are mean ± standard deviation; per month the 18 individuals were pooled by sex: N = 3 pools of female gonads and N = 3 pools of male gonads were established. Within a row, superscripts in capital letters (^ABC^) indicate significant differences between months, asterisk (*) indicate differences between sexes and in lowercase letters (^abc^) indicate significant differences between sex X month (*P* < 0.05).

Σ SFA includes: 12:0, 13:0, iso-14:0, 15:0, iso-15:0, anteiso-15:0, iso-16:0, 17:0, iso-17:0, anteiso-17:0, 22:0, 24:0.

Σ MUFA includes: 14:1n-5, 16:1 n-9, 17:1n-7, 20:1 n-7, 22:1 n-11, 24:1 n-9.

Σ PUFA includes: 16:2n-4, 16:3n-4, 16:4n-1, 18:2n-4, 18:4n-1, 21:5n-3.

### Gonad colour

**Figure 3:**
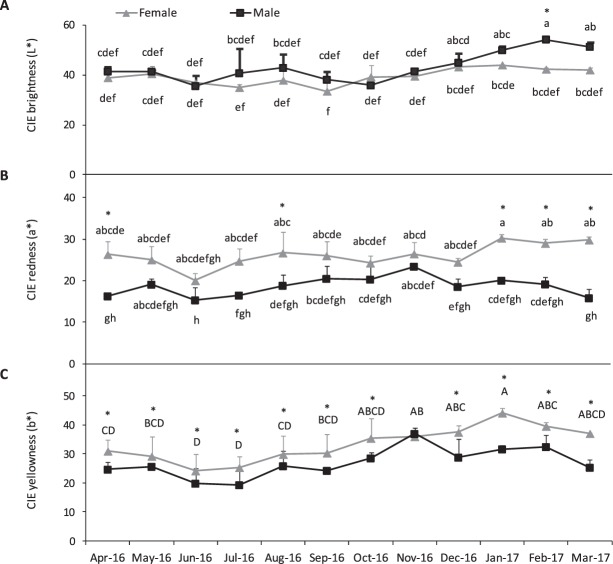
Colour parameters (**A**) lightness, L* (**B**) redness, a* and (**C**) yellowness, b* measured instrumentally in gonads of *P*. *lividus* (ovary and testis) over a 13-month period. Values are mean ± standard deviation; per month the 18 individuals were pooled by sex: N = 3 pools of female gonads and N = 3 pools of male gonads were established were established. Superscripts in capital letters (^ABC^) indicate differences between months, asterisk (*) indicate differences between sexos and superscripts in lowercase letters indicate significant differences between sex X month (^abc^) (P < 0.05).

The seasonal variation of colour parameters lightness (L*), redness (a*) and yellowness (b*) in *P*. *lividus* gonads is presented in Fig. 3. A significant interaction between sex and month factors was found for L* and a* parameters, while b* showed a clear effect of both factors (*P* < 0.05, Supplementary Table S1). Overall, L* values of gonads varied from 33 in September to 54 in February, and only in this month males showed brighter gonads than females (*P* < 0.05, Fig. 3A). Over the sampled period, females had more reddish and yellowish gonads than males, with a* values ranging between 15–30 and b* from 19–44. The highest value of a* and b* was achieved in January, yet levels were also high during gonad maturation period. Lower levels were found during spawning (June-July) for both parameters (Fig. 3B,C). A positive correlation was found for L* and protein content (r = 0.41) as well as for lipid content of gonads and a* and b* (r = 0.75 and 0.72). Also, b* and a* showed to be correlated in gonad (r = 0.77; Table 1).

**Figure 4:**
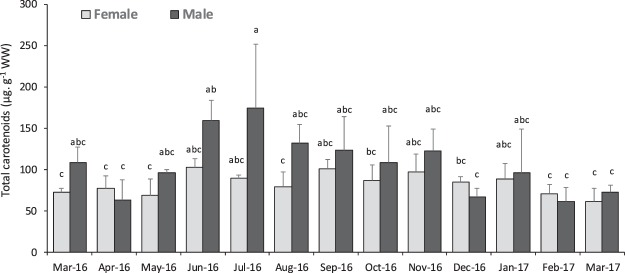
Total carotenoid content in gonads (ovary and testis) of *P*. *lividus* collected at Praia Norte (Portugal) over a 13-month period. Values are mean ± standard deviation; per month the 18 individuals were pooled by sex: N = 3 pools of female gonads and N = 3 pools of male gonads were established. Superscripts in lowercase letters indicate significant differences between sex X months (^abc^) (P < 0.05).

Total carotenoid content in gonads of females and males of *P*. *lividus* is presented in Fig. 4. There was a significant interaction between the effects of sex and month on carotenoid content yet, no differences were found for each factor separately (Supplementary Table S1). Males’ carotenoid content was significantly higher in June-July (159 to 174 µg.g^−1^ WW) during the spawning period when compared to February-March 2017 (61 to 72 µg.g^−1^ WW), months prior to spawning characterized by high GSI values. By contrast, levels of total carotenoids did not vary significantly in females (61 to 103 µg.g^−1^ WW) over the 13-month period. A negative correlation (r = −0.45, Table 1) was found between total carotenoid content and GSI, meaning that higher levels of carotenoids occur during and after spawning.

Five carotenoid pigments were identified and quantified in the gonads of both sexes: α-, β-carotene, echinenone, lutein and β-cryptoxanthin. Levels of individual pigments are shown in Table 3. The gonads of both sexes broadly had similar carotenoid profiles (Supplementary Fig. S1), and echinenone was the dominant carotenoid in gonads, irrespectively of sex. Higher echinenone levels were observed in males during September and November (approx. 171 µg.g^−1^ WW, *P* < 0.05) compared to females. β-cryptoxanthin was the second highest pigment in gonads; it had similar levels in both sexes (*P* ≥ 0.05) and evidenced a seasonal variation (*P* < 0.05, Supplementary Table S1). A slight but significant decrease of β-cryptoxanthin levels occurred in February 2017, from 20.4 to 18.9 µg.g^−1^ WW. Levels of β-carotene were consistently higher than those of α-carotene and did not differ significantly between sex. Between February-April 2016 (before spawning) levels of β-carotene decreased to 10.88 µg.g^−1^ WW, while in September (after spawning) levels peaked up to 26.58 µg.g^−1^ WW. The less abundant pigment in gonads was α-carotene (0.3–2.2 µg.g^−1^ WW), levels were significantly higher in male than in female gonads and peaked in June (during spawning). Females showed significantly higher amount of lutein pigment (0.5–8.7 µg.g^−1^ WW) compared to males (0.9–4.0 µg.g^−1^ WW), and the interaction between sex and month was found significant (*P* < 0.05). Lutein levels in females were higher from April to September, before and after spawning occurred.

**Table 3:** Content of carotenoid pigments found in gonads (ovary and testis, µg.g^−1^ WW) of *P*.

Sex	Female					Male				
Month	Apr-16	Jun-16	Sep-16	Nov-16	Feb-17	Apr-16	Jun-16	Sep-16	Nov-16	Feb-17
**Pigments**										
α-carotene	1.0 ± 0.5^AB^*	1.1 ± 0.7^A^*	0.2 ± 0.02^B^*	0.3 ± 0.2^B^*	0.6 ± 0.2^B^*	1.2 ± 0.7	2.9 ± 1.4	1.8 ± 1.2	0.8 ± 0.5	0.1 ± 0.1
β- carotene	12.3 ± 2.8^B^	14.8 ± 6.2^AB^	19.9 ± 4.6^A^	22.5 ± 7.3^AB^	14.3 ± 5.0^B^	10.8 ± 5.7	19.2 ± 4.9	36.5 ± 19.9	23.1 ± 8.1	7.5 ± 3.3
Echinenone	33.6 ± 4.5^c^	36 ± 17.0^c^	64.1 ± 14.2^c^	93.9 ± 16.8^bc^	75.4 ± 28.7^c^	83.2 ± 37.2^c^	162.3 ± 16.9^ab^	171.6 ± 57.3^a^	170.7 ± 32.6^a^	37.7 ± 7.5^c^
Lutein	8.7 ± 3.9^a^	6.7 ± 1.4^ab^	4.7 ± 0.5^abc^	4.1 ± 1.6^bc^	0.5 ± 0.2^c^	1.0 ± 0.4^c^	1.7 ± 0.6^c^	1.0 ± 0.5^c^	1.0 ± 0.1^c^	4.0 ± 0.7^bc^
β-cryptoxantin	20.1 ± 0.3^A^	18.2 ± 0.04^AB^	19.9 ± 0.5^A^	20.2 ± 0.8^A^	18.7 ± 0.8^B^	20.1 ± 0.7	19.7 ± 0.3	20.4 ± 0.4	20 ± 0.8	19.2 ± 0.4

*lividus* collected at Praia Norte (Portugal) at five distinct periods. Values are mean ± standard deviation; per month the 18 individuals were pooled by sex: N = 3 pools of female gonads and N = 3 pools of male gonads were established. Within a row, superscripts in capital letters (^ABC^) indicate significant differences between months, asterisk (*) indicate differences between sexes and in lowercase letters (^abc^) indicate significant differences between sex X month (*P* < 0.05).

Echinenone pigment was strongly correlated with α- and β-carotene as well as with total carotenoid content (r = 0.60, 0.64 and 0.77, respectively, Table 1). Also, a positive correlation was found between this pigment and protein content of gonads (r = 0.72). Although carotenoids are lipid soluble compounds there was no correlation between echinenone and the lipid content (r = 0.11, *P* = 0.56). β-carotene levels were positively correlated with both total carotenoid and α-carotene content in gonads (r = 0.71 and 0.44). Also, α- carotene was negatively correlated to GSI (r = −0.49, Table 1).

### Gonad texture

Firmness of *P*. *lividus* gonads varied significantly within the three studied months, but remained similar between males and females (Supplementary Table S1). Gonads presented greater firmness in November (33.6–38.4 g) during growing stage (II) than in September (25.3–25.5 g, Table 4), the post-spawn period where most females were in recovery stage (stage I) and more than 50% of males still presented mature or spent gonads, as observed in Fig. 1B. Gonad resilience was not affected over time and was also similar between sexes, ranging from 77.8 to 80.3% (Table 4). Firmness was negatively correlated with moisture (r = −0.63, data not shown) whereas resilience was not correlated with any measured variable.

**Table 4:** Firmness (g) and resilience (%) of *P*. *lividus* gonads (ovary and testis) collected at Praia Norte (Portugal) at three distinct periods.

Sex	Female			Male		
Month	Sep-16	Nov-16	Feb-17	Sep-16	Nov-16	Feb-17
Firmness (g)	25.5 ± 3.7^B^	33.6 ± 8.2 ^A^	31.2 ± 8.7^AB^	25.3 ± 6.7	38.4 ± 3.6	23.3 ± 5.3
Resilience (%)	80.3 ± 0.3	80.0 ± 0.9	79.8 ± 3.2	79.7 ± 1.8	80.0 ± 1.5	77.8 ± 1.4

Values are mean ± standard deviation; per month the 18 individuals were pooled by sex: N = 3 pools of female gonads and N = 3 pools of male gonads were established. Within a row, superscripts in capital letters (^ABC^) indicate significant differences between months (P < 0.05).

## Discussion

Different aspects of the reproductive cycle of *P*. *lividus* were evaluated in this study to fully characterize gonads production, nutritional value, colour and texture in a wild population from the Atlantic coast of Portugal. Our results indicate the occurrence of a single annual spawning event during late Spring and Summer (June-July), coinciding with the rise of seawater temperatures up to its maximum value (16.4 °C). Spirlet, *et al*.^16^ pointed that populations of echinoids from temperate waters exhibit similar reproductive patterns: nutrient storage during Fall and Winter followed by a long spawning season during late Spring and Summer. A single annual spawning season was also described in populations from the Atlantic cost of France, Ireland and Spain^13,16,27^ and in the Mediterranean Sea^28^. However, the occurrence of two spawning events during an annual cycle has been described for *P*. *lividus* in the Mediterranean Sea^29^ and in the central West coast of Portugal^6^, where seawater temperatures are usually higher than those reported in presently studied area.

In this study, the highest GSI value (18%) observed in March 2017 was almost 2.5 times greater than the highest value registered in a southern population (8%) of Portugal^6^. The differences found in the two Portuguese populations might be explained by local environmental conditions, habitat and seasonality factors. According to Ouréns, *et al*.^18^, the GSI values of *P*. *lividus* show a geographical trend, where the Atlantic populations reach higher values than the Mediterranean ones. Also, a latitudinal pattern in the Atlantic populations was described, with higher GSI values at higher latitudes. Environmental cues such as food availability and quality, sea water temperature or/and photoperiod have been suggested to modulate *P*. *lividus* reproductive potential^4^. Interestingly, in this study, the mean GSI value observed at March-16 (9%) varied considerably from that observed in march-17 (17%). Since sea water temperature (mean of 12.5 °C) and day-length (mean of 12 h light) followed a quite similar variation pattern in these particular months, it is possible that changes in food availability or food type could have affected the development and maturation of gonads and thus partially explain the marked variation on GSI values.

GSI showed a seasonal variation, but did not differ between sexes. Similar results were observed in other *P*. *lividus* populations from Portugal^6^, Ireland^13^ and Mediterranean Sea^30^. Sellem and Guillou^31^ highlighted the importance of using histological analysis to interpret GSI variation; these authors related punctual decreases of GSI values with changes in gonad reserves rather to gamete release. However, changes in the GSI observed over the study period, namely gonad size and weight, followed the gonad maturation pattern described by histological analysis: an increase of size was observed during the growing (II) stage due to nutrient storage in the non-germinal cells, continued until reaching the maximum yield at maturation (IV) stage then, with spawning, gonads became smaller and empty in content (stages V and VI).

The biochemical composition of *P*. *lividus* gonads showed a clear seasonal variation also varying with sex. Protein was the major component of gonads, ranging between 12–18% WW while total lipid content was considered low (≤6% WW). All biochemical parameters were within the range of values previously reported for *P*. *lividus*^27,28,32^. Moreover, the seasonal variation of nutrient content in gonads reflected the reproductive cycle. Previous reports showed that the best period to harvest gonads with high nutritional quality and commercial value is during the growing period, generally prior to the onset of gametogenesis, when the nutritive phagocytes are full^33^ and accumulate higher levels of protein, lipid and carbohydrates^34^. In the present study we observed that gonads reached their highest content of protein, lipid and energy in November (growing stage, II) and maintained this high nutritional value until January-February (pre-maturation stage, III), before full maturation of gametes, suggesting this period as desirable for harvesting nutrient-rich gonads. Afterwards, when gonads were mature and peaked their GSI (March-17), their nutritive content started decreasing, in terms of lipids and energy, reinforcing that gonad quality deteriorates with gametes development. Changes in the biochemical content of gonads can impair their flavour, leading to a loss of economic value. Murata, *et al*.^35^ described that mature ovaries of *Hemicentrotus pulcherrimus* had a bitter taste, which was strongly correlated with the presence of a novel sulfur-containing amino acid, pulcherrimine. Furthermore, it was shown that the yolk protein stored in nutritive phagocytes is used during gametogenesis to synthesize new proteins and other nitrogen substances^19^, which might contribute to changes in gonad flavour. Despite these observations, no correlation could be found between the biochemical components of gonad and the GSI (which reflected gametogenic stages), confirming previous observations by Montero-Torreiro and Garcia-Martinez^27^. Likewise in many other species, the nutritional composition of edible tissues (gonads) seems to be highly dependent on sea urchin diet^36^. Changes on feed type and abundance throughout the year may affect the assimilation of nutrients and modify gonad composition. Moreover, recent studies have evaluated the effect of natural (algae) and formulated diets on *P*. *lividus* gonad biochemical composition^32,37,38^ in order to promote the production of high valued gonads.

The FA profile of *P*. *lividus* gonads was in general agreement with data reported in the literature^28,38–40^. But only a few of these studies discern sex as factor, mostly focusing on the effects of season or diet. Martínez-Pita, *et al*.^39^ found differences in the FA profile of ovary and testis of *P*. *lividus:* females showed higher levels of 14:0, LA and ALA whereas males exhibited higher levels of 18:0 and ARA; in terms of FA classes, only SFA differed between sexes, being higher in females. These authors also observed differences in the FA profile of two distinct populations, which suggested that environmental factors, such as food source, may influence the gonad FA profile of individuals of the same sex. The source and availably of food, as well as other site-associated factors, should be taken into consideration when results are being compared with the literature, since it may mask the effects of sex or season. In our work, females showed much higher concentrations of PUFA, MUFA and SFA compared to males, and although concentrations were within the expected values for sea urchins, the variation between sexes was more pronounced compared to other studies of *P*. *lividus*^39^ and *Psammechinus miliaris*^41^.

To our knowledge, this is the first time that effects of sex/season/gametogenic cycle on FA composition of *P*. *lividus* male and female gonads, has been presented for a wild population. The seasonal effect on FA composition of gonads was clear; females and males had preferentially higher concentrations of the sum of PUFA, MUFA and SFA classes in November, which was coincident with the period of gonad growth (stage II), when the gonads were richer in nutrients. It was possible to relate the season factor with the gametogenic cycle, yet no correlation was found between GSI and any FA in gonads. Carboni, *et al*.^37^ showed that the FA profile of *P*. *lividus* (without sex distinction) changed during gametogenesis, however differences were related to dietary lipid intake and not to seasonality. As previously discussed, diet has an important role in determining the nutritional composition of gonads^36^. Sea urchins are generalist grazers, but they often prefer feeding on large kelp (*Laminaria* ps.) species. The selective ingestion of certain macro algae and their respective nutritional value, have certainly contributed to modulate the FA content of sea urchin gonads once macro algae are rich in PUFA, particularly in EPA^42^. A recent study conducted in the North coast of Portugal with *Laminaria ochroleuca*, an algae consumed by *P*. *lividus*^4^, showed a seasonal pattern of growth and reproduction for this algae^43^. Interestingly, *L*. *ochroleuca* accumulated more nutrients during its growth period (winter)^43^, which was coincident with the increase of nutrient content of gonads, namely FA.

Regarding the most relevant PUFA, EPA was preferentially accumulated in the ovary and levels decreased with increasing maturity stage, from September-November to February 2017. Previous studies have reported a trend of EPA accumulation in the gonads of sea urchins^37,41^. In contrast, ARA and DHA levels only suffered a seasonal variation, being more concentrated in November, in both males and females. The recommended daily consumption of EPA + DHA to decrease the risk of cardiovascular diseases in humans is between 0.25-0.5 g^44^. If sea urchins are consumed during the period of highest concentration of PUFA in gonads (the growing stage in November) an intake of 50 g of *P*. *lividus* (around 5 adults) can provide near 0.245 g of EPA + DHA, which covers the daily recommended dose to promote human health, reinforcing the nutritional importance of sea urchin.

Few studies have evaluated the seasonal variation of gonad colour from wild populations of sea urchins, despite the relevance of this trait for market valorisation. In most cases gonad colour was evaluated visually or instrumentally and without sex differentiation^36,45,46^. The results obtained by the CIE L*a*b* system were within the range of values reported for wild *P*. *lividus* fed on macroalgae. Seasonal changes in gonad colour were also reported for *Strongylocentrotus franciscanus*^12^ and *S*. *droebachiensis*^1^, which may be related to changes in gonad yield and possibly maturation stage (gametogenesis). In the present study, a positive correlation between L*, b* values and the GSI was observed, suggesting that the increase of gonad yield, from the growing to the full maturation stage, led to increased brightness and yellowish colour. Moreover, previous findings in *P*. *lividus* showed that gonads during the spawning season had an unacceptable coloration (dark red/brown) whilst during the growing period exhibited an excellent/good coloration (bright orange or yellow)^45^. In terms of colour, Cook and Kelly^47^ classified as acceptable to excellent coloration (pale yellow to bright orange) gonads presenting (mean) values of 42 for L*, 20 for a* and 35 for b*. Our results showed that L* a* and b* values observed, in both sexes, between November (growing stage) and February (pre-mature stage) were similar or higher than those proposed by Cook and Kelly^47^, suggesting that gonads presented a desirable coloration for market. Additionally, it was also in this period that gonads become nutritionally richer and increased in size, as previously discussed. By combining all these positive features, one can propose the period between November and February as the most suitable to harvest premium gonads. Moreover, colour values decreased at spawning (June), showing some loss of colour quality, as previously observed with the nutrient content.

Total carotenoids in gonads did not differ between sexes but showed some seasonality. Total carotenoid content varied with gonad maturation stages and followed a similar pattern in several sea urchin species, increasing during spawning (June) probably due to gonad loss of biomass, and decreasing afterwards. This is consistent with previous observations in *P*. *lividus*^45^, *P*. *miliaris*^48^ and *S*. *droebachiensis*^49^. Carotenoids are pigments deposited in the gonads however, and according to Symonds, *et al*.^45^, acceptability of gonad colour is not directly linked neither with the levels of individual carotenoids nor their ratios found in the gonad. In fact total carotenoids picked after spawning, when gonads are known to be unacceptable for consumers^45^.

Echinenone was the dominant carotenoid in the gonad of both males and females, confirming previous reports for this species^20,45^. Moreover, echinenone levels were highest in males after spawning and onset of recovery period (September-November) whereas females showed more steady levels over time. Although no detailed evaluation of carotenoids deposition in gametes (eggs and semen) was performed, the white colour of semen contrasts with the orange coloration of eggs, suggesting that males might have kept most of the carotenoids in their gonads during spawning, that could partly explain their highest concentration after spawning, when gonad volume is strongly reduced. Moreover, males showed a considerable variation of echinenone concentration after spawning, which was also observed by Symonds, *et al*.^45^. This is probably related with the high variability in gametogenic stages of males observed post-spawning, that were found at recovery, partly spawned and spent stages. Also, no correlation could be found between echinenone in gonads and L* a* b* parameters, confirming previous observations in *P*. *lividus*^20,45^ and *P*. *miliaris*^48^. Gonads present a high content of pigments but its biochemical nature failed to be correlated with data obtained by instrumental or sensorial analysis, suggesting that the visual perception of colour may be influenced by other factors, besides carotenoid pigments. In *P*. *lividus*, unacceptable gonad coloration was observed with both very low and high levels of echinenone and total carotenoids in gonads^45^. These results suggest that carotenoids clearly made a contribution towards the red-orange pigmentation of the sea urchin gonad, but other factors may induce the sensorial evaluation of colour. The second major group of pigments present *in P*. *lividus* gonads were β-carotene and β-cryptoxantin, which only differ seasonally and not between sex. These findings may reinforce the hypothesis that, in *P*. *lividus*, echinenone is likely to be formed by bio-conversion of β-carotene via β-isocryptoxanthin^10^ and its metabolism may occur mainly in gonads^21^. Symonds, *et al*.^45^ also identified β-carotene as the second major group of pigments in *P*. *lividus* gonads, but in a different study, Shpigel, *et al*.^20^ reported the absence of β-carotene in gonads of urchins fed with a natural algal diet.

Firmer gonads were observed in November, during the growing period (stage II), whereas in both sexes, lower gonad firmness was observed in September (after spawning), when gonads appear as spent. Firmness was negatively correlated with gonad moisture content (r = −0.63), being in agreement with McBride, *et al*.^12^. These authors demonstrated that *S*. *franciscanus* gonads had higher moisture content and softer texture during maturation whereas the opposite was observed during the recovering and growing period, suggesting that gonads firmness was increased by lower moisture content. Few studies determined gonad texture, and were mostly focused on subjective evaluations^23,50^ rather than in instrumental analysis using a texture analyser^12^. The effect of sex in gonad texture was never reported before. Resilience is considered an useful texture measure for market valorisation, since a strong relation between high resilience values and high-quality rated gonads was reported by McBride, *et al*.^12^. In the present study, gonads resilience was not affected over time nor differed between sexes, being always higher than 75%. This may suggest that resilience remains acceptable throughout the year cycle.

In conclusion, this study clearly demonstrated that most of the evaluated quality attributes were mainly dependent on sea urchin sex, but also showed a seasonal variation. According to the present observations, it is suggested that the best period for commercial harvesting sea urchins in the North Atlantic coast of Portugal is between November and February. During this period, gonads are mainly at the growing and premature stages of gametogenesis, holding a good nutrient storage in their nutritive phagocytes, as demonstrated by the highest levels of protein, lipid, n-3 and n-6 PUFA and energy content, in both males and females. In addition, gonads showed lower moisture content and simultaneously higher firmness, that is a valuable attribute. Also during this period, gonads were found more reddish, yellowish and brighter, irrespectively of the sex, being the values within those considered acceptable and desirable in the European market, were a bright and yellowish-orange gonad is rated premium. Moreover, as it was evidenced that most commercially important traits change seasonally compromising the gonad value, sea urchin production under controlled conditions (echinoculture) can be a sustainable alternative to assure a regular supply of high quality gonads. The present data can be further used to help tailoring new formulated feeds able to produce sea urchins with the desired gonad attributes for international markets.

## Material and Methods

### Ethics statement

All animal procedures respecting capture of wild specimens and tissue sampling were previously approved by the CIIMAR ethical committee, in compliance with the European Union Directive 2010/63/EU and the Portuguese law Decreto-Lei n⍛ 113/2013 on “protection of animals used for scientific purposes”. Wild sea urchins *P. lividus* were collected at Praia Norte (41°41′57″N, 8°51′13″W), Portugal, after authorization from the national maritime authority (Captaincy of the Port of Viana do Castelo) and the Portuguese Institute for Nature Conservation and Forests (ICNF). Moreover, the sampling site did not involve protected areas nor endangered/protected species in the Atlantic region, and followed the Portuguese law Portaria n.⍛ 82/2011 on “minimum size for captures and harvesting”, implying that only commercial sized sea urchins (test diameter ≥5 cm) were collected.

### Animals and sampling

Adult *P*. *lividus* were collected between March 2016 and March 2017, by snorkeling in subtidal rocky reefs of Praia Norte, Portugal. Sea temperature records from A Guarda (Spain) oceanic buoy (station nearby our sampling area; 41°54′16.8″N, 8°53′51.0″W) were obtained from CMEMS (http://marine.copernicus.eu-SST_GLO_SST_L4_NRT_OBSERVATIONS) through AquaSafe by Hidromod (http://www.hidromod.com). Day length data were obtained for Porto city (near our sampling site) and assessed from the Observatório Astronónico de Lisboa, University of Lisbon^26^.

**Figure 5:**
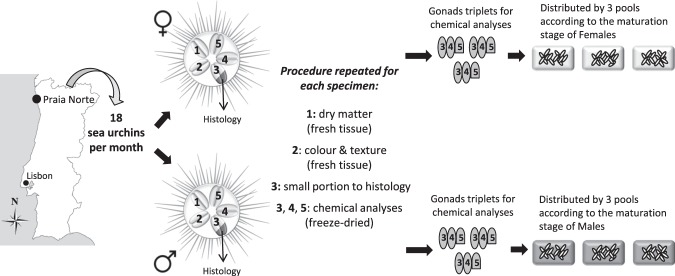
Summary of the monthly sampling protocol performed for *P*. *lividus* at Praia Norte (Portugal) over a 13-month period.

Eighteen (N = 18) sea urchins were collected monthly and transported to CIIMAR facilities in seawater. Individual records of test diameter and total wet weight were performed in laboratory; animals were then placed in an ice bath for 30 min before dissection (Fig. 5). The five gonads of each animal were carefully removed, damp-dried and weighted for individual gonad somatic index (GSI) determination, calculated as: [(g gonad wet weight/g total wet weight) x 100]. The sex of the specimens was identified by direct observation of released gametes from the dissected gonads and later confirmed by histological examination. One gonad was used for instrumental assessment of colour and texture and another one for dry matter determination (Fig. 5). A small gonad sample was immediately fixed in 4% buffered formalin for 24 h and the remaining gonads were individually snap-frozen and kept at −80 °C for subsequent biochemical analyses (Fig. 5). Fixed gonad samples were dehydrated, embedded in paraffin and transversely sectioned at 7μm. Sections were stained with hematoxylin-eosin and observed under a binocular microscope (CX22LED, Olympus). The gametogenic stages of ovary and testes were identified according to Byrne^13^: recovery (stage I), with primary gametes and nutritive phagocytes; growing (stage II) with clusters of primary gametes and packed nutritive phagocytes, premature (stage III) with gametes at all stages of development and reduced amount of nutritive phagocytes; mature (stage IV) with mature gametes and few nutritive phagocytes; partly spawned (stage V) with loosely packed gametes and depletion of nutritive phagocytes and spent (stage VI) with gonads empty of gametes.

It is not possible to distinguish externally the sex of *P*. *lividus*, likewise other sea urchin species, yet both male and female individuals were collected monthly over the 13-month period, without needing any further sampling during the same month. Sex ratio in samplings varied over time: a minimum of seven and maximum of eleven individuals from the same sex were collected (sex ratio of 7:11, N = 18), but a ratio of 9:9 female:male (N = 18) was found in the majority of the sampled months (Fig. 5). Due to the small size of gonads, individual samples were pooled monthly by sex: three pools of female gonads (N = 3) and three pools of male gonads (N = 3) were established. Individuals from the same sex were pooled based on their maturation stage, after confirmation by histological analysis, as previous studies on *P*. *lividus* have related changes on nutritional composition and carotenoid content of gonads with their maturation stage^27,45,51^. The number of individuals in a pool was variable and dependent on the sex ratio and maturation stage of gonads, yet the three pools of male and three pools of female gonads were always ensured per month. Before analyses, gonad pools were quickly crushed in liquid nitrogen and reduced to a fine powder without defrosting. Half of this frozen sample was used for carotenoids analysis, whereas the rest was freeze-dried before all other biochemical analyses.

### Biochemical composition

Chemical analyses were run in duplicate, following the AOAC^52^ procedures. Fresh gonads were analysed for dry matter after drying at 105 °C for 24 h, and samples of freeze-dried gonads were analysed for: ash content by incineration in a muffle furnace at 500 °C for 6 h (Nabertherm L9/11/B170, Germany); crude protein (N x 6.25) by a flash combustion technique followed by a gas chromatographic separation and thermal conductivity detection (LECO FP428, USA) and gross energy in an adiabatic bomb calorimeter (IKA C2000, Germany). Total lipids were determined following the method described by Folch, *et al*.^53^ with dichloromethane-methanol (2:1) and gravimetric determination.

The fatty acid methyl esters (FAME) contained in total lipid extracts were transesterified by acidic methylation^54^, as described by Campos, *et al*.^55^. To each sample was added 1 mL of internal standard solution (1 mg C23:0/1 mL hexane; C23:0, Matreya LLC, USA). FAME were analysed in duplicate, using a Shimadzu GC-2010 Plus gas chromatograph (Shimadzu Europe GmbH, Germany), equipped with a flame-ionization detector and an Omegawax 250 capillary column (30 m x 0.25 mm i.d. x 0.25 µm film thickness; Supelco, Bellefonte, USA). FAME were identified by comparing their retention times with known standards and quantified as mg.g^−1^ of dry biomass, using the internal standard C23:0.

### Colour determination

Gonad colour was evaluated instrumentally and by determination of the carotenoid content and profile. Colour was measured by the CIE 1976 (L*, lightness: a*, redness; b*, yellowness) method using a CR-400 colorimeter (Konica Minolta) at standard illuminant D_65_. Readings were calibrated against a white tile and three replicate measurements were taken for each gonad and averaged to determine colour parameters.

Total carotenoid extraction was performed according to Symonds, *et al*.^45^ with some modifications. Briefly, carotenoids from frozen gonad tissue (0.4–0.5 g) were extracted, in duplicate, in acetone (5:1, v:w). After centrifugation at 3500 × g for 10 min, at 8 °C, the supernatant was collected and the extraction procedure was repeated twice to obtain a colourless supernatant. Supernatants were evaporated to dryness under a constant stream of nitrogen, re-suspended in n-hexane and filtered with a 0.2 μL syringe filter for further analysis. Total carotenoid content was determined in a spectrophotometer (Shimadzu UV-1603, Japan), using the absorption maximum in hexane and the extinction coefficient (E^1%^_1cm_) of 2500^56^, based in the equation describe by Pocock, *et al*.^57^:

$${\rm{C}}=({\rm{E}}\times {\rm{V}}\times 1000)/[100\times {\rm{G}}\times ({{{\rm{E}}}^{1 \% }}_{1{\rm{cm}}})]$$

where, C = concentration of carotenoid (mg)/wet weight (g). E = maximal absorption of a known volume of carotenoid solution read at the wavelength of maximum absorption. V = sample final volume (ml). E^1%^_1cm_ = the extinction coefficient of a 1% (w/v) solution in a 1 cm cuvette at a defined wavelength. G = sample weight (g).

The qualitative and quantitative profile of carotenoids was performed by high-performance liquid chromatography analysis (Waters Alliance Series 600, Mildford, USA), and was restricted to five sampled months: April, June, September, November 2016 and February 2017, selected to cover the gametogenic stages identified in this study. Each carotenoid extract was suspended in n-hexane and 20 μL were injected in a reverse phase Acclaim™ C30 LC column (250 × 4.6 mm i.d., 5μm particle size, Thermo Scientific) with a mixture of acetonitrile, methanol, dichloromethane, hexane and ammonium acetate (55:22:11.5:11.5:0.02, v-v:v:v:w) under isocratic conditions (1.0 mL/min, 25 min at 25 °C). Detection was achieved by a diode array detector (Waters 996 PDA, USA) at 454 nm. Retention times and spectra of compounds were analysed by comparison with pure standards and quantification performed by the calibration curves of α- carotene, β-carotene, echinenone, (Sigma, Portugal), lutein and β-cryptoxanthin (Extrasynthése, France). Data were analysed using the Waters Empower™ 2 software and expressed as mg.g^−1^ of gonad biomass.

### Texture evaluation

An instrumental evaluation of firmness and resilience properties was performed in gonads using a TA.XT.plus analyser, fitted with a 5 kg load cell and controlled by Exponent v6 software (Stable Micro systems, UK). Firmness was defined as the force (g) required to compress a sample at 25% of its original height (at a 1.7 mm/s) using a 35 mm cylinder probe. Resilience was defined as the capacity of a sample to recover its original height after a compression with the same probe, being determined as: Resilience (%) = (post-test height/original height) × 100. Briefly, after firmness determination the gonad was compressed up to 60% of its original height and held that position for 2 s. The post-test height was recorded after 15 s. Tests were performed individually in fresh gonads samples in three months of the studied period – September, November and February – which represented the recovery (I), growing (II) and premature (III) stages of gametogenesis, respectively. These stages of gametogenesis were selected based on their relevance to gonad quality, since market acceptability is higher before gametes development and spawning.

### Statistical analysis

Data are presented as means and standard deviation. Tests for normality and homogeneity of variances were performed by Kolmogorov-Smirnov and Levene’s tests, respectively. A two-way ANOVA followed by HSD Tukey test, using sex (female and male) and month as independent variables, was performed with STATISTICS 6.4 package (StatSoft, Inc., Tulsa, OK, USA). Pearson’s correlation was used to relate all parameters, independently of the sex factor. In all cases significant differences were considered when *P* < 0.05.

## Supplementary information

Dataset 1

## References

[1] Robinson SMC, Castell JD, Kennedy EJ (2002). Developing suitable colour in the gonads of cultured green sea urchins (*Strongylocentrotus droebachiensis*). Aquaculture.

[2] FAO. *Fisheries and aquaculture software*. *Global Fisheries commodities production and trade 1976-2016*. *FishstatJ – software for fishery stastical time series*., http://www.fao.org/fishery/statistics/software/fishstatj/en (2018).

[3] Micael, J., Alves, M. J., Costa, A. C. & Jones, M. B. In *Oceanography and marine biology* Vol. 47 (eds R. N. R. N. Gibson, R. J. A. Atkinson, & J. D. M. Gordon) 191–208 (CRC Press, 2009).

[4] Boudouresque, C. F. & Verlaque, M. In *Sea urchins: Biology and ecology* Vol. 38 (ed. John M. Lawrence) Ch. 21, 297–327 (Elsevier, 2013).

[5] FAO. Food and Agriculture Organization Fisheries Statistics. (2016).

[6] Gago, J., Range, P. & Luis, O. J. In *Echinoderm Research 2001* (eds Féral, J. P. & David, B.) 269–276 (A.A. Balkema, 2003).

[7] INE. Statistics Portugal. Nominal catch (t) by landed port and specie – 2015, https://www.ine.pt/xportal/xmain?xpid=INE&xpgid=ine_indicadores&indOcorrCod=0001073&contexto=bd&selTab=tab2 (2016).

[8] Bertocci I (2014). Multiple effects of harvesting on populations of the purple sea urchin *Paracentrotus lividus* in north Portugal. Fisheries Research.

[9] Sun, J. & Chiang, F. S. In *Echinoderm Aquaculture* (eds Nicholas, P. Brown & Stephen, D. Eddy) Ch. 2, 25–45 (John Wiley & Sons, Inc, 2015).

[10] Kelly, M. S. & Symonds, R. C. In *Sea Urchins: Biology and Ecology* Vol. Volume 38 (ed M. Lawrence John) 171–177 (Elsevier, 2013).

[11] Phillips K (2010). Effect of manufactured diets on the yield, biochemical composition and sensory quality of *Evechinus chloroticus* sea urchin gonads. Aquaculture.

[12] McBride SC, Price RJ, Tom PD, Lawrence JM, Lawrence AL (2004). Comparison of gonad quality factors: color, hardness and resilience, of *Strongylocentrotus franciscanus* between sea urchins fed prepared feed or algal diets and sea urchins harvested from the Northern California fishery. Aquaculture.

[13] Byrne M (1990). Annual reproductive cycles of the commercial sea urchin *Paracentrotus lividus* from an exposed intertidal and a sheltered subtidal habitat on the west coast of Ireland. Marine Biology.

[14] Walker, C. W., Lesser, M. P. & Unuma, T. In *Sea Urchins: Biology and Ecology* Vol. Volume 38 (ed. M. Lawrence John) Ch. 3, 25–43 (Elsevier, 2013).

[15] Shpigel M, McBride SC, Marciano S, Lupatsch I (2004). The effect of photoperiod and temperature on the reproduction of European sea urchin *Paracentrotus lividus*. Aquaculture.

[16] Spirlet C, Grosjean P, Jangoux M (1998). Reproductive cycle of the echinoid *Paracentrotus lividus*: analysis by means of the maturity index. Invertebrate Reproduction & Development.

[17] Spirlet C, Grosjean P, Jangoux M (2000). Optimization of gonad growth by manipulation of temperature and photoperiod in cultivated sea urchins, *Paracentrotus lividus* (Lamarck) (Echinodermata). Aquaculture.

[18] Ouréns R, Fernández L, Freire J (2011). Geographic, population, and seasonal patterns in the reproductive parameters of the sea urchin *Paracentrotus lividus*. Marine Biology.

[19] Unuma T, Yamamoto T, Akiyama T, Shiraishi M, Ohta H (2003). Quantitative changes in yolk protein and other components in the ovary and testis of the sea urchin *Pseudocentrotus depressus*. Journal of Experimental Biology.

[20] Shpigel M, Schlosser SC, Ben-Amotz A, Lawrence AL, Lawrence JM (2006). Effects of dietary carotenoid on the gut and the gonad of the sea urchin *Paracentrotus lividus*. Aquaculture.

[21] Shpigel M, McBride SC, Marciano S, Ron S, Ben-Amotz A (2005). Improving gonad colour and somatic index in the European sea urchin *Paracentrotus lividus*. Aquaculture.

[22] Haug E, Guillou M, Connan S, Goulard F, Diouris M (2003). HPLC analysis of algal pigments to define diet of sea urchins. Journal of the Marine Biological Association of the United Kingdom.

[23] Azad AK, Pearce CM, McKinley R (2011). Effects of diet and temperature on ingestion, absorption, assimilation, gonad yield, and gonad quality of the purple sea urchin (*Strongylocentrotus purpuratus*). Aquaculture.

[24] Rocha AC (2018). Bioaccumulation of persistent and emerging pollutants in wild sea urchin *Paracentrotus lividus*. Environmental research.

[25] Bertocci I, Dominguez R, Freitas C, Sousa-Pinto I (2012). Patterns of variation of intertidal species of commercial interest in the Parque Litoral Norte (north Portugal) MPA: comparison with three reference shores. Marine environmental research.

[26] Observatorio Astronomico Lisboa, O. A. L. *Astronomical data - Porto*, http://oal.ul.pt/publicacoes/almanaques/dados-de-2017/, http://oal.ul.pt/publicacoes/almanaques/dados-de-2016/ (2018).

[27] Montero-Torreiro MF, Garcia-Martinez P (2003). Seasonal changes in the biochemical composition of body components of the sea urchin, *Paracentrotus lividus*, in Lorbé ((Galicia, north-western Spain). Journal of the Marine Biological Association of the United Kingdom.

[28] Arafa S, Chouaibi M, Sadok S, El Abed A (2012). The Influence of Season on the Gonad Index and Biochemical Composition of the Sea Urchin *Paracentrotus lividus* from the Golf of Tunis. The Scientific World Journal.

[29] Fernandez C (1998). Seasonal Changes in the Biochemical Composition of the Edible Sea Urchin *Paracentrotus lividus* (Echinodermata: Echinoidea) in a Lagoonal Environment. Marine Ecology.

[30] Lozano, J. *et al*. Biological cycles and recruitment of *Paracentrotus lividus* (Echinodermata: Echinoidea) in two contrasting habitats. *Marine Ecology Progress Series*, 179–191 (1995).

[31] Sellem F, Guillou M (2007). Reproductive biology of *Paracentrotus lividus* (Echinodermata: Echinoidea) in two contrasting habitats of northern Tunisia (south-east Mediterranean). Journal of the Marine Biological Association of the United Kingdom.

[32] Tenuzzo, B. A., Carata, E., Mariano, S. & Dini, L. In *Sea Urchin - From Environment to Aquaculture and Biomedicine* (ed. Maria Agnello) 73–91 (InTech, 2017).

[33] Walker, C. W. *et al*. In *Echinoderm Aquaculture* (eds Nicholas Brown & Stephen, D. Eddy) Ch. 12, 263–286 (John Wiley & Sons, Inc, 2015).

[34] Marsh, A. G., Powell, M. L. & Watts, S. A. In *Sea Urchins: Biology and Ecology* Vol. 38 (ed John M. Lawrence) 45–57 (Elsevier, 2013).

[35] Murata Y (2002). Seasonal changes of bitterness and pulcherrimine content in gonads of green sea urchin Hemicentrotus pulcherrimus at Iwaki in Fukushima Prefecture. Fisheries Science.

[36] Lourenço, S., Valente, L. M. P. & Andrade, C. Meta‐analysis on nutrition studies modulating sea urchin roe growth, colour and taste. *Reviews in Aquaculture*, 1–16, 10.1111/raq.12256 (2018).

[37] Carboni S, Hughes AD, Atack T, Tocher DR, Migaud H (2013). Fatty acid profiles during gametogenesis in sea urchin (*Paracentrotus lividus*): Effects of dietary inputs on gonad, egg and embryo profiles. Comparative Biochemistry and Physiology Part A: Molecular & Integrative Physiology.

[38] Volpe MG (2018). Gonad quality of sea urchin *Paracentrotus lividus* cultured in an offshore pilot‐scale trial on the south‐east Italian coast. Aquaculture Nutrition.

[39] Martínez-Pita I, García FJ, Pita M-L (2010). Males and females gonad fatty acids of the sea urchins *Paracentrotus lividus* and *Arbacia lixula* (Echinodermata). Helgoland Marine Research.

[40] Angioni A, Addis P (2014). Characterization of the lipid fraction of wild sea urchin from the Sardinian Sea (western Mediterranean). Journal of food science.

[41] Hughes AD, Kelly MS, Barnes DKA, Catarino AI, Black KD (2006). The dual functions of sea urchin gonads are reflected in the temporal variations of their biochemistry. Marine Biology.

[42] Dawczynski C, Schubert R, Jahreis G (2007). Amino acids, fatty acids, and dietary fibre in edible seaweed products. Food Chemistry.

[43] Pereira TR, Azevedo IC, Oliveira P, Silva DM, Sousa-Pinto I (2019). Life history traits of Laminaria ochroleuca in Portugal: The range-center of its geographical distribution. Aquatic Botany.

[44] EFSA. In *EFSA Journal* Vol. 8 1461 (Wiley-Blackwell, 2010).

[45] Symonds RC, Kelly MS, Caris-Veyrat C, Young AJ (2007). Carotenoids in the sea urchin *Paracentrotus lividus*: occurrence of 9′-cis-echinenone as the dominant carotenoid in gonad colour determination. Comparative Biochemistry and Physiology Part B: Biochemistry and Molecular Biology.

[46] Pearce CM, Daggett TL, Robinson SMC (2004). Effect of urchin size and diet on gonad yield and quality in the green sea urchin (*Strongylocentrotus droebachiensis*). Aquaculture.

[47] Cook EJ, Kelly MS (2007). Enhanced production of the sea urchin *Paracentrotus lividus* in integrated open-water cultivation with Atlantic salmon *Salmo salar*. Aquaculture.

[48] Symonds RC, Kelly MS, Suckling CC, Young AJ (2009). Carotenoids in the gonad and gut of the edible sea urchin *Psammechinus miliaris*. Aquaculture.

[49] Hagen, N. T., Jorgensen, I. & Egeland, E. S. *Sex-specific seasonal variation in the carotenoid content of sea urchin gonads*. Vol. 3 (2008).

[50] Phillips K (2009). Effect of gender, diet and storage time on the physical properties and sensory quality of sea urchin (*Evechinus chloroticus*) gonads. Aquaculture.

[51] Mol S, Baygar T, Varlik C, Tosun SY (2008). Seasonal variations in yield, fatty acids, amino acids and proximate compositions of sea urchin (*Paracentrotus lividus*) roe. Journal of Food and Drug Analysis.

[52] AOAC. *Official methods of analysis of AOAC International*. 18 edn, (AOAC International, 2006).

[53] Folch J, Lees M, Stanley GHS (1957). A simple method for the isolation and purification of total lipids from animal tissues. Journal of Biological Chemistry.

[54] Lepage G, Roy CC (1986). Direct transesterification of all classes of lipids in a one-step reaction. Journal of lipid research.

[55] Campos I, Matos E, Marques A, Valente LMP (2017). Hydrolyzed feather meal as a partial fishmeal replacement in diets for European seabass (*Dicentrarchus labrax*) juveniles. Aquaculture.

[56] Tsushima M, Byrne M, Amemiya S, Matsuno T (1995). Comparative biochemical studies of carotenoids in sea urchins—III. Relationship between developmental mode and carotenoids in the Australian echinoids *Heliocidaris erythrogramma* and *H*. *tuberculata* and a comparison with Japanese species. Comparative Biochemistry and Physiology Part B: Biochemistry and Molecular Biology.

[57] Pocock T, Krol M, Huner NP (2004). The determination and quantification of photosynthetic pigments by reverse phase high-performance liquid chromatography, thin-layer chromatography, and spectrophotometry. Methods in molecular biology.

