# Supplementary material for: The effect of sex, season and gametogenic cycle on gonad yield, biochemical composition and quality traits of Paracentrotus lividus along the North Atlantic coast of Portugal
Source: Sci Rep. 2019 Feb 28;9:2994. doi: 10.1038/s41598-019-39912-w (PMC6395811; doi:10.1038/s41598-019-39912-w)
Supplement: Supplementary file 1 — Dataset 1 [file 41598_2019_39912_MOESM1_ESM.docx]

**The effect of sex, season and gametogenic cycle on gonad yield, biochemical composition and quality traits of *Paracentrotus lividus* along the North Atlantic coast of Portugal**

Filipa Rocha^1^, Luís F. Baião^1,2^, Sara Moutinho^1^, Bruno Reis^1,2^, Ana Oliveira^3^, Francisco Arenas^1^, Margarida R.G. Maia^4^, António J. M. Fonseca^2,4^, Manuela Pintado^3^ and Luisa M. P. Valente^1,2*^

^1^ CIIMAR/CIMAR, Interdisciplinary Centre of Marine and Environmental Research, University of Porto, Terminal de Cruzeiros do Porto de Leixões, Av. General Nórton de Matos, S/N, 4450-208 Matosinhos, Portugal.

^2^ ICBAS, Abel Salazar Biomedical Sciences Institute, University of Porto, Rua Jorge Viterbo Ferreira 228, 4050-313 Porto, Portugal.

^3^ CBQF, Faculty of Biotechnology, Portuguese Catholic University, Rua Dr. António Bernardino de Almeida, 4200-072 Porto, Portugal.

^4^ REQUIMTE, LAQV, ICBAS, Abel Salazar Biomedical Sciences Institute, University of Porto, Rua de Jorge Viterbo Ferreira 228, 4050-313 Porto, Portugal.

* Corresponding author: phone: (+351) 223 401 825; e-mail: [lvalente@icbas.up.pt](mailto:lvalente@icbas.up.pt)

**Supplementary material**

**Table S1.** *P*-values obtained after a two-way ANOVA analyses of parameters assessed in *P. lividus*. Main factors were sex (female and male) and month (13-month period from March 2016 to March 2017, except for carotenoid pigments and texture). Sex X Month represents the interaction between the two factors. Values were considered significantly different for *P˂*0.05. Carotenoid pigments determination and texture evaluation were performed at a 5- and 3-month periods, respectively.

**Figure S1.** HPLC chromatogram showing the carotenoid profile of a) female and b) male *P. lividus* gonad. The pigments were identified as: (1) lutein; (2) echinenone; (3) β-cryptoxanthin; (4) α-carotene and (5) β-carotene.

 **A B**

**1**

**3**

**2**

**4**

**5**

**2**

**3**

**5**

**4**

**1**

**1**
